# Serum under-*O*-glycosylated IgA1 level is not correlated with glomerular IgA deposition based upon heterogeneity in the composition of immune complexes in IgA nephropathy

**DOI:** 10.1186/1471-2369-15-89

**Published:** 2014-06-13

**Authors:** Kenji Satake, Yoshio Shimizu, Yohei Sasaki, Hiroyuki Yanagawa, Hitoshi Suzuki, Yusuke Suzuki, Satoshi Horikoshi, Shinichiro Honda, Kazuko Shibuya, Akira Shibuya, Yasuhiko Tomino

**Affiliations:** 1 Division of Nephrology, Department of Internal Medicine, Juntendo University Faculty of Medicine, 2-1-1 Hongo, Bunkyo-ku, Tokyo 113-8421, Japan; 2 Department of Immunology, Institute of Basic Medical Sciences, Faculty of Medicine, University of Tsukuba, Ibaraki, Japan

**Keywords:** Under-*O*-glycosylated IgA1, Glomerular IgA deposition, Decision tree analysis

## Abstract

**Background:**

Although serum under-*O*-glycosylated IgA1 in IgA nephropathy (IgAN) patients may deposit more preferentially in glomeruli than heavily-*O*-glycosylated IgA1, the relationship between the glomerular IgA deposition level and the *O*-glycan profiles of serum IgA1 remains obscure.

**Methods:**

Serum total under-*O*-glycosylated IgA1 levels were quantified in 32 IgAN patients by an enzyme-linked immunosorbent assay (ELISA) with *Helix aspersa* (HAA) lectin. Serum under-*O*-glycosylated polymeric IgA1 (pIgA1) was selectively measured by an original method using mouse Fcα/μ receptor (mFcα/μR) transfectant and flow cytometry (pIgA1 trap). The percentage area of IgA deposition in the whole glomeruli (Area-IgA) was quantified by image analysis on the immunofluorescence of biopsy specimens. Correlations were assessed between the Area-IgA and data from HAA-ELISA or pIgA1 trap. The relationships between clinical parameters and data from HAA-ELISA or pIgA1 trap were analyzed by data mining approach.

**Results:**

While the under-*O*-glycosylated IgA1 levels in IgAN patients were significantly higher than those in healthy controls when measured (p < 0.05), there was no significant difference in under-*O*-glycosylated pIgA1. There was neither a correlation observed between the data from HAA-ELISA and pIgA1 trap (r^2^ = 0.09) in the IgAN patients (r^2^ = 0.005) nor was there a linear correlation between Area-IgA and data from HAA-ELISA or the pIgA1 trap (r^2^ = 0.005, 0.03, respectively). Contour plots of clinical parameters versus data from HAA-ELISA and the pIgA1 trap revealed that patients with a high score in each clinical parameter concentrated in specific areas, showing that patients with specific *O*-glycan profiles of IgA1 have similar clinical parameters. A decision tree analysis suggested that dominant immune complexes in glomeruli were consisted of: 1) IgA1-IgG and complements, 2) pIgA1 and complements, and 3) monomeric IgA1-IgA or aggregated monomeric IgA1.

**Conclusions:**

Serum under-*O*-glycosylated IgA1 levels are not correlated with glomerular IgA deposition based upon heterogeneity in the composition of glomerular immune complexes in IgAN patients.

## Background

IgA nephropathy (IgAN), the most common glomerulonephritis and the major cause of end-stage kidney disease worldwide, is characterized by the presence of IgA1 deposits in glomerular mesangial areas [1,2]. IgAN patients display three major alterations in IgA1: an increased level of circulating polymeric IgA1 (pIgA1), IgA containing immune complexes [3-6], and under-*O*-glycosylation in the hinge region [2,7].

The presence of abnormal *O*-glycans is related to the self-aggregation of IgA1 [8,9]. The exposed terminal *N*-acetyl-*D*-galactosamine (GalNAc), resulted from under-*O*-glycosylation, presents novel epitopes recognized by IgG and IgA auto-reactive antibodies [9-11]. Although it has been thought that under-*O*-glycosylated IgA1 can deposit in glomeruli more preferentially, the correlation between the amount of serum under-*O*-glycosylated IgA1 and its glomerular deposition has not been clarified.

A lectin from *Helix aspersa* (HAA), which recognizes GalNac, has been used to develop an enzyme-linked immunoadsorbent assay (HAA ELISA) for the measurement of galactose-deficient IgA1 in sera [7]. However, the selective analysis of *O*-glycans in the hinge region of pIgA1, which play an important role in the formation of immune complexes and glomerular deposition, is difficult since under-*O*-glycosylated monomeric and pIgA1 are simultaneously measured by this method. We developed a novel method to quantify under-*O*-glycosylated pIgA1 using a mouse Fcα/μ receptor (mFcα/μR) transfectant (pIgA1 trap). Therefore, mFcα/μR transfectants were used for the detection of human pIgA1 in the present study. Fcα/μ R is a high affinity Fc receptor for IgA and IgM. Its gene is located close to the polymeric immunoglobulin receptor (poly-IgR) on chromosome 1 [12,13]. Fcα/μR binds to pIgA, but not to monomeric IgA because the ligand polymerization status is crucial for the interaction of Fcα/μR [14,15]. Purified IgG bindings, irrespective of subclasses and aggregation, were not observed with the Fcα/μR transfectant [16].

In this study, the correlation between serum under-*O*-glycosylated whole IgA1 or pIgA1 levels in IgAN patients and glomerular IgA deposition in IgAN patients was assessed.

## Methods

### Patients

Sera were obtained from 32 patients with IgAN within 10 days prior to renal biopsy and diagnosed by the presence of dominant IgA1 deposits in glomerular mesangial areas and mesangial cell proliferation. Sera from 20 healthy controls were also studied. All patients and controls were older than 18 years of age at the time of blood sampling for this study. The levels of serum creatinine (s-Cr), IgA, complement 3 (C3), and random spot urinary protein/Cr ratio were determined. The number of red blood cells (RBCs) was identified in urinary sediment and the severity of hematuria was graded as grade 1 [1–5 RBCs/high-power field (HPF)], grade 2 (6–20 RBCs/HPF), or grade 3 (≥21 RBCs/HPF) [17]. The estimated glomerular filtration rate (eGFR) was calculated using the formula established by the Japanese Society of Nephrology for Japanese people: 194 × s-Cr^−1.094^ × age^−0.287^ (×0.739 if female) [18]. The serum IgA concentration of healthy controls was measured by ELISA.

This study was approved by Ethics Review Committee of Juntendo University Faculty of Medicine and complied with the Helsinki Declaration 1975. Written informed consent was obtained from all patients.

#### pIgA1 trap analysis

The mouse T cell leukemic cell-line BW5147 transfectant stably expressing mFcα/μR has been described previously [12]. In our preliminary study, it was confirmed that the affinity of mFcα/μR for human pIgA1 was much stronger than that of human Fcα/μR. The alternatively spliced variants of hFcα/μR have been reported [13,14], UniProtKB; Q8WWV6, FCAMR_HUMAN] and the cDNA which we used for constructing hFcα/μR transfectants had shorter N-terminal leader sequences than that of mFcα/μR [12]. Although the amino acid sequences of immunoglobulin binding site of h-and mFcα/μR have been highly conserved, those around this domain are different [12].

**Figure 1:**
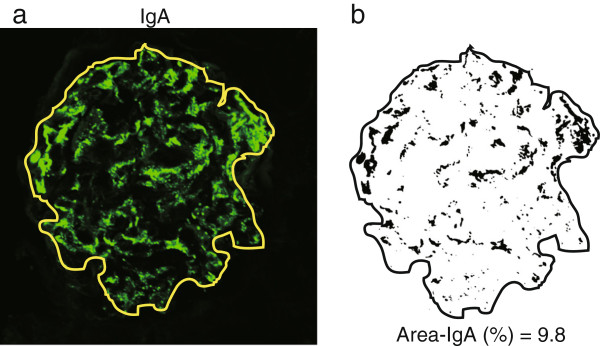
**Area measurement of IgA deposition in a glomerulus.** As an example, an immunofluorescence image of a renal biopsy specimen from an IgAN patient is shown. **a**. The brightness of the immunofluorescence photograph was adjusted by image J software and the edge of glomerulus was traced. **b**. The color photograph was converted to binary data and the edge of glomerulus was traced. Area-IgA (%) was calculated by the following formula. Area-IgA (%) = (black pixel number) / (whole pixel number within the traced area) × 100. The Area-IgA was 9.8%.

For a negative control, mock transfectant cells were exposed to Plat E packaging cell-line producing the pMX-neo vector [19]. The mFcα/μR expressing transfectant (Fcα/μR transfectant) and parent BW5147 cells (2 × 10^5^) were treated with 5 μl of sera from IgAN patients or from healthy controls at 4°C for 30 min. The cells were washed three times with an FACS staining buffer [0.5% phosphate buffered saline, 0.05% (w/v) bovine serum albumin (BSA), and 0.05% (w/v) sodium azide]. The sodium azide was added to an FACS staining buffer to immobilize the cytoskeleton of the cells in good viability since mFcα/μR internalizes into cytosol when it is cross-linked by ligands [12]. The transfectant and parent cells were stained with biotinylated *Helix aspersa* (HAA) lectin (Sigma-Aldrich, St. Louis, MO, USA) at 4°C for 15 min. After washing three times, the transfectant and parent cells reacted with 1:500 diluted phycoerythrin (PE)-conjugated streptavidin (Beckman Coulter, CA, USA) at 4°C for 30 min. Dead cells were excluded by propidium iodide (PI) staining (Figure 1a). The mean fluorescence intensity (MFI) of each cell was measured by flow cytometry (FACS Calibur, Becton Dickinson, NJ, USA). Before starting assay, mFcα/μR transfectant and parent cells were preliminary treated with serum in the same protocol and stained by a PE-conjugated mouse monoclonal anti-human IgA antibody (Miltenyl Biotec, Clone IS11-8E10 (isotype: mouse IgG1, intact molecule), Bergisch Gladbach, Germany) to confirm that the transfectant cells were saturated by IgA1 and that the parent cells were not non-specifically stained. The amount of pIgA1 analyzed in each procedure was estimated around 10 μg using purified pIgA1 (Data not shown). Biotinylated mouse monoclonal anti-human IgM antibody (Miltenyi Biotec, Clone PJ2-22H3 (isotype: mouse IgG1, intact molecule)) was also used when IgM bindings to the cells were checked.

Secretory IgA purified from pooled human colostrum using multistep procedures which may include salt fractionation, gel filtration, ion-exchange chromatography, and immunoadsorption (MP Biomdicals, Santa Ana, CA, USA) were adopted as a positive control of the pIgA1 trap. Human monomeric IgA1 and pIgA1 from multiple myeloma patients and degalactosylated pIgA1 were kindly provided by Professor Jan Novak (University of Alabama at Birmingham, AL, USA) and used as controls.

#### HAA ELISA

HAA lectin was used to determine serum IgA1 with aberrantly *O*-glycosylation, as reported previously [11,20]. For capture ELISA, flat-bottom, 96-well plates were coated at 4°C overnight with F(ab’)2 fragment of goat anti-human IgA antibody (Jackson ImmunoResarch Laboratories, West Grove, PA, USA) at a concentration of 2.5 μg/ml. The plates were blocked at 4°C overnight with 1% BSA in PBS containing 0.05% Tween 20 (v/v). Samples diluted in blocking buffer were added to each well and incubated at 4°C overnight. The captured IgA1 was subsequently desialylated by treatment at 37°C for 3 hours with 10 mU/ml neuraminidase from *Vibrio cholera* (Roche Applied Science, Indianapolice, IN, USA) in 10 mM sodium acetate buffer (pH5). Samples were then incubated at 37°C for 3 hours with biotinylated HAA lectin (Sigma-Aldrich) diluted in the blocking buffer. The bound lectin was detected with an avidin-horseradish peroxidase conjugate. The peroxidase chromogenic substrate *o*-phenylenediamine-H_2_O_2_ (Sigma-Aldrich) was then added. The color reaction was stopped with 1 M sulfuric acid and the absorbance at 490 nm was measured using Spectra Max (Molecular Devices, Sunnyvale, CA, USA). The HAA reactivity of IgA1 in each sample was then calculated as OD units/1 μg of IgA1. Naturally degalactosylated IgA1 purified from the plasma of a patient with IgA1 multiple myeloma was treated with neuraminidase and used as a standard [7,21]. For comparisons of HAA bindings, the OD units per 1 μg standard degalactosylated IgA1 were assigned a value of 100% and it was used as the HAA ELISA titer.

#### Immunofluorescence of renal biopsy sections and measurement of IgA deposition area in glomerulus (Area-IgA)

Staining for IgG, IgA, and C3 in glomeruli on freshly frozen renal sections (3 μm) was performed using corresponding fluorescein isothiocyanate (FITC)-conjugated anti-sera (Dako, Copenhagen, Denmark). The samples were analyzed under a confocal laser-scanning microscope (Fluoview FV1000, Olympus, Tokyo, Japan) and the electronic images were stocked in the JPEG format.

The image files were analyzed by Image Processing and Analysis in Java software (Image J, NIH, Bethesda, MD, USA). The brightness of the immunofluorescence images were adjusted to a range of 25–255 pixel values and the edges of glomeruli were traced to delimit glomerular areas (Figure 1a). The color (RGB) immunofluorescence images were converted to binary data and pixels in black and within whole traced (glomerular) areas were counted using the *Analyze Particle* command. IgA deposition areas (Area-IgA,%) were calculated by the following formula (Figure 1b):

$$\begin{array}{ll} \mathrm{Area}-\mathrm{IgA}\left(\%\right)= & \left(\mathrm{black}\;\mathrm{pixel}\;\mathrm{count}\right)/\\ & \left(\mathrm{whole}\;\mathrm{pixel}\;\mathrm{count}\;\mathrm{within}\;\mathrm{the}\;\mathrm{traced}\;\mathrm{area}\right)\\ & \times 100 \end{array}$$

IgG and C3 deposition areas (Area-IgG, Area-C3, respectively) (%) were also calculated using the same procedure.

#### Colocalization assay of HAA lectin and IgA1 on mFcα/μR transfectant

The colocalization of HAA lectin and IgA1 on the mFcα/μR transfectant was analyzed by confocal laser-scanning microscope. The cells were treated with pIgA1 for 15 min, washed 3 times, and incubated with an FITC-conjugated rabbit anti-human IgA (DAKO) antiserum at 1:100 dilution. After washing, the cells were incubated again with biotinylated HAA lectin at room temperature for 2 hours. Floating cells were adhered to slides using cytospin (Thermo Scientific, Waltham, MA, USA) and post-staining with DAPI was also performed. Double staining studies with human IgA and IgM were also performed to the mFcα/μR transfectant previously treated with human sera using an FITC-conjugated anti-human IgA and biotinylated anti-human IgM antisera which was detected by DyLight 549 conjugated streptavidin (Jackson ImmunoResarch Laboratories). The cells were also fixed to slides by cytospin and DAPI staining was performed.

#### Renal histological grading

The renal histological grade was determined using the criteria of the Joint Committee of Research Groups on Progressive Renal Diseases (Ministry of Health, Labor and Welfare of Japan) and Japanese Society of Nephrology [22].

#### Statistics

All values were examined by mean ± SD. Differences between the two groups were evaluated using a Mann-Whitney’s U test. Comparisons among three or more parameters were analyzed by analysis of variance. P < 0.05 was defined as statistical significant. Statistical analyses, including contour plots, in which data from pIgA1 trap form the X-axis, data from ELISA form the Y axis, and other clinical parameters form a pseudo-Z axis via colors (age, interval between onset and biopsy, Area-IgA, Area-IgG, Area-C3, serum IgA, serum C3, the IgA/C3 ratio, s-Cr, eGFR, and urinary protein excretion), were performed using JMP7 (SAS Institute, Cary, NC, USA).

To verify Area-IgA in glomerulus, a decision tree analysis was used with the HAA ELISA titer and pIgA1 trap value by JMP7. Decision tree algorithms aim to divide the data set into subsets that give the best discrimination between groups. Each subset (called a *node* in a decision tree) can split the data set into subsets to sharpen the discrimination between groups. A 10-fold cross validation was performed by WEKA (Waikato Environment for Knowledge Analysis).

## Results

### Characteristics of IgAN patients and healthy controls

Participating IgAN patients consisted of 11 males and 21 females with a mean age of 30.3 ± 8.3 years. Mean s-Cr and eGFR were 0.81 ± 0.28 mg/dl and 75.5 ± 20.5 ml/min/1.73 m^2^, respectively. Urinary protein excretion and serum C3 levels were 1.0 ± 1.3 g/g Cr and 98 ± 13.5 mg/dl, respectively. There was no significant difference in the gender distribution between IgAN patients and healthy controls. The age was significantly lower and serum IgA level was significantly higher in IgAN patients than in healthy controls (p < 0.01 and p < 0.001, respectively) (Table 1).

**Table 1:** Characteristics of IgAN patients and healthy controls

	**Normal range**	**IgAN (n = 32)**	**Healthy control (n = 20)**	**Difference**
Gender (M/F)		11/21	12/8	
Age (yrs)		30.3 ± 8.3	35.1 ± 4.0	p < 0.01
IgA (mg/dl)	110–410	305.5 ± 127.1	177.8 ± 64.6	p < 0.001
Creatinine (mg/dl)	0.6–1.0	0.81 ± 0.28	Not done	
eGFR (ml/min/1.73 m^2^)		75.5 ± 20.5	Not done	
Urinary protein (g/g Cr)		1.0 ± 1.3	Not done	
C3 (mg/dl)	69–128	98 ± 13.5	Not done	

#### pIgA1 trap, a novel pIgA1 specific *O*-glycan analysis

**Figure 2:**
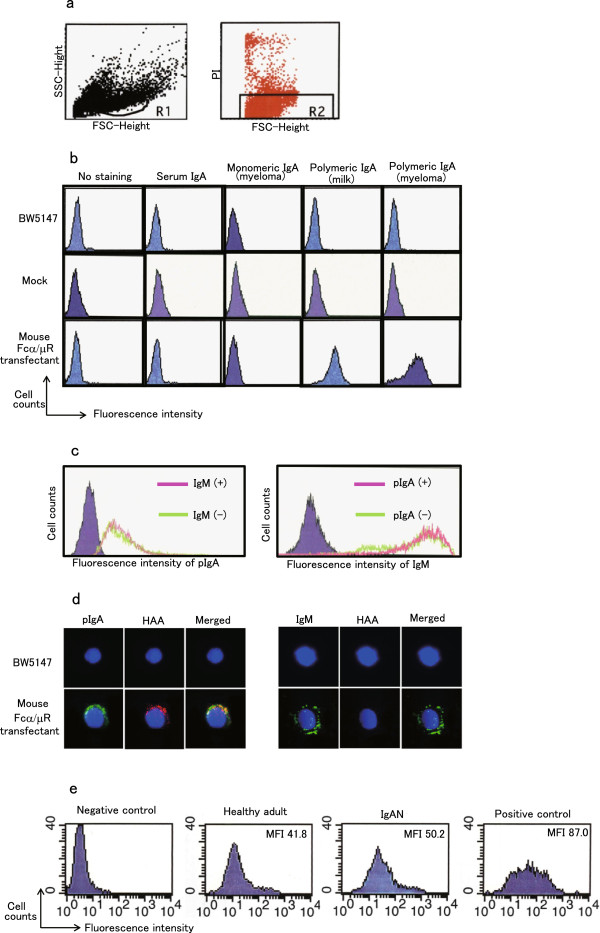
**pIgA1 trap, a novel pIgA1 specific for *O*-glycan analysis.** Serum pIgA1 was captured by mouse Fcα/μR transfectant and *O*-glycans of captured pIgA1 were stained with fluorescein-labeled HAA lectin. The fluorescence intensity of the transfectant was detected by flow cytometry. **a**. Dead cells were extracted from analysis. **b**. Mouse Fcα/μR transfectant can bind to pIgA1 from both human milk and myeloma serum but not bind mIgA1. **c**. IgM does not interfere in pIgA1 binding to mouse Fcα/μR. Mouse Fcα/μR transfectant was pretreated with or without human IgM. pIgA1 was added to these cells and pIgA1 binding on cell surface was detected by PE-conjugated anti-human IgA. IgM had no effect for pIgA1 binding to mouse Fcα/μR transfectant (left panel). Similarly, pretreatment with pIgA1 did not interfere IgM binding to mouse Fcα/μR (right panel). **d**. HAA lectin only binds to pIgA1 but not IgM. Mouse Fcα/μR transfectant was pretreated with pIgA1. Confocal images collected from one mouse Fcα/μR transfectant triply stained for anti-human IgA antibody (pIgA1, green), degalactosylated *O*-glycans (HAA lectin, red), DAPI (nucleus, blue), and their merged images. A similar procedure was performed for BW5147 (parent) cells as a negative control. **e**. pIgA1 trap analysis. Mouse Fcα/μR transfectant was pretreated with human serum and washed. The cells were stained with biotin-conjugated HAA and PE-conjugated streptavidin. The healthy adult was a 44 year-old male and the IgAN patient was a 19 year-old female. A negative control was prepared without serum and a positive control was prepared with pIgA1 from myeloma which has been known as highly degalactosylated by ELISA.

Serum pIgA was trapped using mouse Fcα/μR transfectant. The *O*-glycans of the captured pIgA1 were stained with fluorescein-labeled HAA lectin and the fluorescein intensity of the tranfectant was measured by flow cytometry. Dead cells were distinguished from the flow cytometric study by the measurement of a combination of forward scatter (FSC), side scatter (SSC), and propidium iodide (PI) staining (Figure 2a). Purified serum IgA (monomeric IgA) and monomeric IgA1 from multiple myeloma patients didn’t bind to the BW5147 parent cell, the mock transfectant, or the mFcα/μR transfectant. Both pIgA1 from milk and multiple myeloma patients tightly bound to the mFcα/μR transfectant but showed no reactivity to BW5147 or the mock transfectant (Figure 2b). The mFcα/μR transfectant pre-treated with IgM revealed similar binding activity of pIgA1 to non-treated transfectant and showed the same binding activity with or without pre-treatment with IgM (Figure 2c). While pIgA1 bound mFcα/μR transfectant can fix HAA, the IgM bound mFcα/μR transfectant could not react with HAA. The merged figures revealed the co-localization of pIgA1 and HAA, suggesting that HAA bound to under-glycosylated *O*-glycan of pIgA1 (Figure 2d). Serum pIgA1 was captured by mFcα/μR transfectant and was followed by staining with fluorescein labeled HAA. The fluorescence intensity of the HAA-bound transfectant could be measured and it varied in each patient or healthy control. The positive control, which was performed with degalactosylated pIgA1 from multiple myeloma patients, showed a higher intensity of fluorescence of HAA (Figure 2e).

#### HAA ELISA and pIgA1 trap

**Figure 3:**
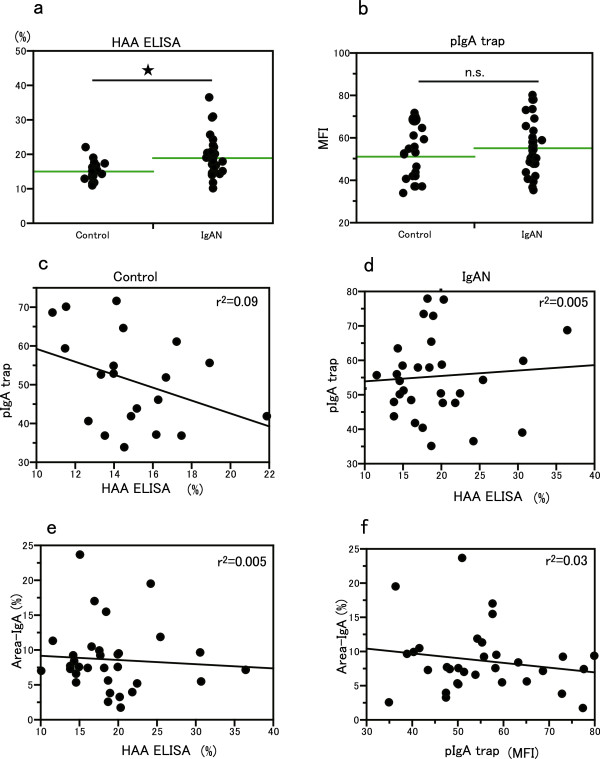
**HAA ELISA and pIgA1 trap. a**. HAA ELISA between healthy control and IgAN. There was significantly more HAA ELISA titer in the IgAN patients than in healthy controls (p < 0.05). **b**. pIgA1 trap between healthy control and IgAN. There was no significant difference of pIgA1 data (MFI) between IgAN patients and healthy controls. **c**,**d**. Correlation between pIgA1 trap and HAA ELISA. There was no inverse relation between pIgA1 trap data (MFI) and HAA ELISA titers in healthy controls (r^2^ = 0.09, P = 0.11) (Figure 3c) and no direct relation in IgAN patients (r^2^ = 0.005, P = 0.14) (Figure 3d). **e**. Correlation between Area-IgA and HAA ELISA. There was no significant correlation between Area-IgA and HAA ELISA titer (r^2^ = 0.005, P = 0.69). **f**. Correlation between Area-IgA and pIgA1 trap. There was no inverse relation between Area-IgA and pIgA1 trap data (MFI) (r^2^ = 0.03, P = 0.33).

HAA ELISA, which was classically used for the measurement of under-glycosylated *O*-glycan of serum IgA1, was performed for IgAN patients and healthy controls. Similar to previous reports [7,10,11,21], the mean ELISA titer of IgAN patients (19.0 ± 5.7%, mean ± SD) was significantly higher than that of the healthy controls (15.0 ± 2.7%, P < 0.05) (Figure 3a). pIgA1 trap was also performed for the same samples and it revealed that there was no significant difference in the mean value of pIgA1 trap between IgAN patients and healthy controls (Figure 3b). There was no correlation between the values of HAA ELISA and pIgA1 trap, even in healthy controls (r^2^ = 0.09, p = 0.11) (Figure 3c) or in IgAN patients (r^2^ = 0.005, P = 0.14) (Figure 3d). In IgAN patients, a direct correlation was not observed between Area-IgA and HAA ELISA values (r^2^ = 0.005, p = 0.69) (Figure 3e). Similarly, Area-IgA was not directly correlated with the values of pIgA1 (MFI) (r^2^ = 0.03, p = 0.33) (Figure 3f).

#### Contour plot analysis

**Figure 4:**
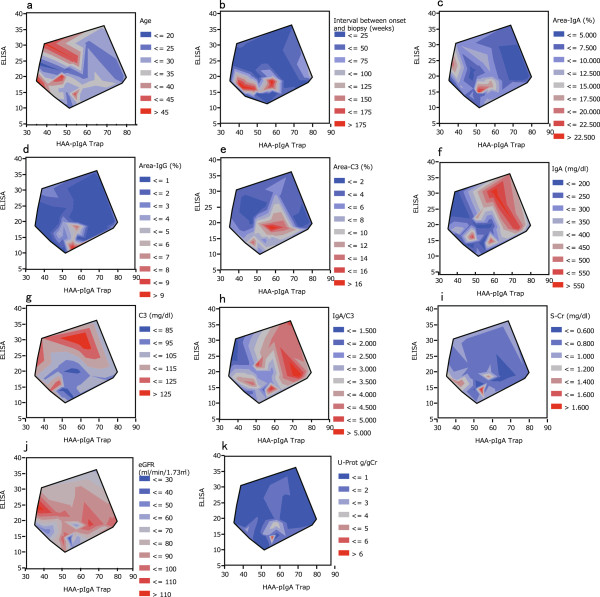
**Contour plots showing the clinical parameters (contour lines and colors) versus the data from pIgA1 trap and HAA-ELISA.** Data from pIgA1 trap form the X-axis, data from ELISA form the Y axis, and the following clinical parameters form a pseudo-Z axis via colors: age **(a)**, interval between onset and biopsy **(b)**, Area-IgA **(c)**, Area-IgG **(d)**, Area-C3 **(e)**, serum IgA **(f)**, serum C3 **(g)**, IgA/C3 ratio **(h)**, s-Cr **(i)**, eGFR **(j)**, and urinary protein excretion **(k)**.

Contour plots showed that the patients with high values in clinical parameters concentrated in specific areas in each plot (Figure 4a–k). The patients with high Area-IgA (over 15%) formed a group in the area prescribed by HAA ELISA titers between 15 and 20 and HAA-pIgA1 trap between 50 and 60 (Figure 4c). The patients in this area showed a long interval between onset and biopsy and a higher score of Area-IgG, Area-C3, s-Cr and urinary protein excretion (Figures 4b,d,e,i,and k, respectively). Inversely, these patients showed low values of serum IgA, serum C3, and eGFR (Figures 4f,g, and j, respectively). Area-IgA and serum IgA as well as Area-C3 and serum C3 showed a contrasted pattern (Figures 4c,f,e,g). In the zone of around 20–25 in HAA ELISA titers, younger age patients and high eGFR patients were concentrated (Figures 4a and j).

#### Decision tree analysis

**Figure 5:**
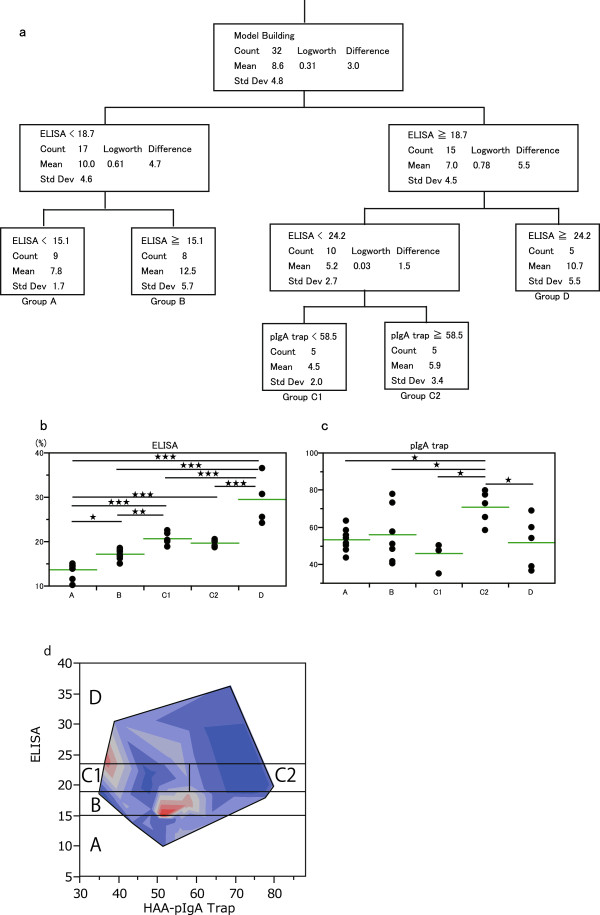
**Decision tree analysis. a**. Decision tree from IgAN patients cohort (n = 32). The decision tree consists of data from HAA ELISA (ELISA) and pIgA1 trap based rules and 8 daughter nodes. Each node provides the data from HAA ELISA or a pIgA1 trap based rule, total number of IgAN patients (Count), mean Area-IgA (%) (Mean), and its standard deviation (Std Dev). The Logworth values are the logs of adjusted p-values for a chi-square test of independence. The nodes are constructed so as to maximize the separation of the two groups, as measured by the sum of squares due to differences between means (Difference). The patients belong to each terminal node grouped as A, B, C1, C2, and D. **b**. HAA ELISA values in each group. HAA ELISA values in the patients belonging to terminal nodes A, B, C1, C2, and D are shown. The horizontal bar shows the mean value of each group. ^★^P < 0.05, ^★★^P < 0.01, ^★★★^P < 0.001 (The stars shows the difference in the data from both ends of a line). **c**. pIgA1 trap values in each group. Data from pIgA1 trap (MFI values) in the patients belonging to each group (terminal node) are shown. ^★^P < 0.05, ^★★^P < 0.01, ^★★★^P < 0.001 (The stars shows the difference in the data from both ends of a line). **d**. The area of each group divided by decision tree analysis on the contour plot of Area-IgA.

Since Area-IgA was not linearly correlated with the values from HAA ELISA and contour plots suggested that there was a complex rule between Area-IgA and under-glycosylated *O*-glycan of serum IgA1, a decision tree analysis was performed to predict Area-IgA in glomeruli and to classify IgAN patients using a combination of HAA ELISA and pIgA1 trap. The decision tree consisted of 5 terminal nodes and patients who belonged to each node were classified into 5 groups (Group A, B, C1, C2, and D) (Figure 5a). Group A was classified by a HAA-ELISA titer value of <15.1% with Group B characterized by an HAA-ELISA titer value of ≥15.1% and <18.7%. Group C was classified by an HAA-ELISA titer value of ≥18.7% and <24.2% and this group was the only group sub-classed into C1 and C2 by pIgA1 trap value (MFI) with a cut off value of 58.5. Group D was classified by a HAA-ELISA titer value of ≥24.2% (Figure 5a).

While the mean value of each HAA-ELISA titer group was significantly different, except for that of C1 and C2 (Figure 5b), the pIgA1 trap value in Group C2 was significantly higher than the other groups and there was no difference between any pairs of Groups A, B, C1, and D (Figure 5c). The obtained model showed a 90.6% accuracy evaluated using a 10-fold cross validation. The overlaid borders settled by decision tree on the contour plot of Area-IgA indicated that patients with a high score of Area-IgA belonged to Group B (Figure 5d).

#### Immunoglobulin or complement deposition area in glomeruli and classified groups

**Figure 6:**
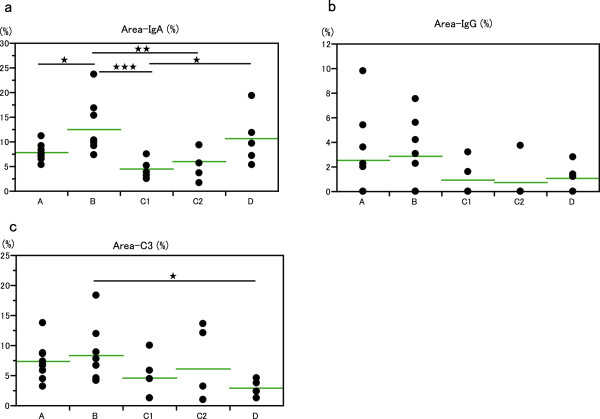
**Immunoglobulin and complement deposition area in glomerulus. a**. Distribution of Area-IgA (%) in each terminal node (group). Area-IgA (%) of each patient is plotted and the difference of each group was estimated. The horizontal bar shows the mean value of the group. ^★^P < 0.05, ^★★^P < 0.01, ^★★★^P < 0.001 (The stars shows the difference in the data from both ends of a line). **b**. Distribution of IgG deposition area (%) in each group. There was no significant difference of IgG deposition area between any pairs of each group. **c**. Distribution of C3 deposition area (%) in each group. The C3 deposition area in glomerulus (%) of each patient is plotted as IgA and IgG. The mean value of Group D was significantly lower than that of Group B (^★^P < 0.05).

A model of Area-IgA was built by decision tree analysis. While the HAA-ELISA titer was increasing from Group A to D, the mean value of Area-IgA showed a different distribution. Although the mean value of Area-IgA in Group B was significantly higher than that in Group A, lower values were found groups C1 and C2. The mean value of Area-IgA increased again in Group D, over those in groups C1 and C2, and there was no difference in the value of Area-IgA between groups B and D (Figure 6a). The mean value of Area-IgG in groups A and B was higher than that of groups C1, C2, and D but there was no significant difference (Figure 6b). The mean value of Area-C3 in glomerulus in groups A and B was more than that in C1, C2 and C3, and a significant difference was observed between groups B and D (Figure 6c).

#### Clinical parameters and classified groups

**Figure 7:**
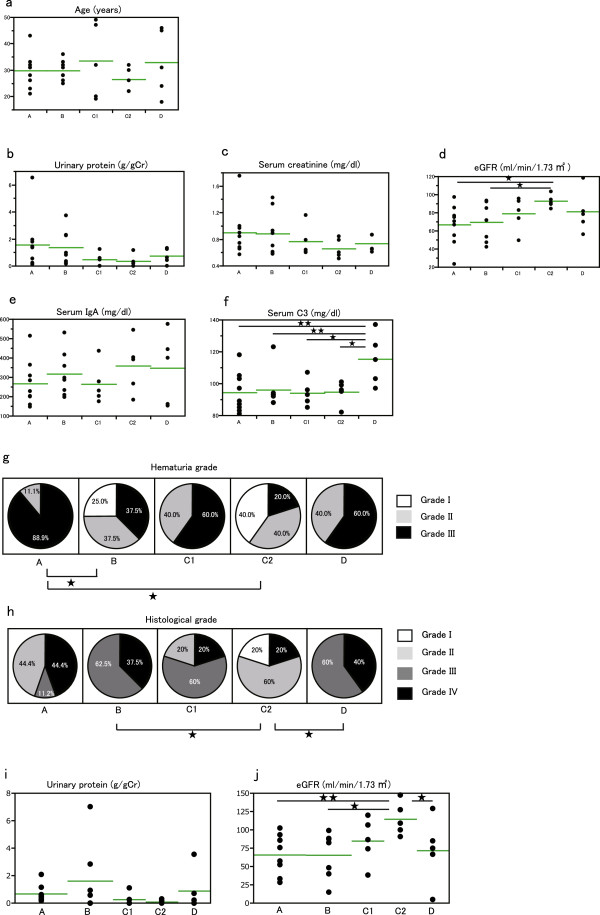
**Clinical parameters in each group. a**. Age distribution. **b**. Distribution of urinary protein. **c**. Distribution of s-Cr. **d**. Distribution of eGFR. The mean eGFR is significantly larger than in Group C2 than in groups **B** and C1 (^★^P < 0.05). **e**. Distribution of serum IgA. **f**. Distribution of serum C3. Mean value of serum C3 in group **D** is significantly more than all other groups. (^★^P < 0.05, ^★★^P < 0.01). **g**. Distribution of hematuria grade. The percent distribution of the hematuria grade in each group is shown. The patients in group A reveal significantly more severe hematuria than those in group A and C2 (^★^P < 0.05). **h**. Distribution of renal historical grade. The percentage of the historical grade in each group is shown. The histological grade of renal specimens of groups **B** and **D** were significantly worse than those of group C2 (^★^P < 0.05). **i**. Distribution of urinary protein excretion three years after renal biopsy. **j**. Distribution of eGFR three years after renal biopsy. The mean eGFR of group C2 is significantly higher than that of group **A**, **B** and **D** (^★^P < 0.05, ^★★^P < 0.01).

Since it was suggested that the IgAN patients classified by decision tree analysis using HAA ELISA titer and pIgA1 trap values were also clinically different, clinical parameters were compared. The mean patients’ age in Group C2 seemed younger than other groups but there was no significant difference (Figure 7a). Urinary protein excretion (g/g Cr) and s-Cr (mg/dl) showed a similar distribution in that groups A and B were in a higher range, groups C1 and C2 in a lower range, and Group D in a middle range, although these values had no significant difference (Figure 7b, 7c). Mean eGFR in Group C2 was significantly higher than that in groups A and B. Mean eGFR values in groups C1 and D were in the middle range and showed no significant difference with Group C2 (Figure 7d).

No significant difference in the mean serum IgA level in each group was observed (Figure 7e). The mean serum C3 level in Group D was significantly more elevated than in all the other groups (Figure 7f). The patients in Group A revealed a more severe hematuria than those in groups B and C2 (Figure 7g). The histological grade of renal specimens in groups B and D were significantly more severe compared with that of Group C2 (Figure 7h).

The urinary protein excretion level was not significantly different three years after renal biopsy (Figure 7i). The mean eGFR of group C2 was significantly higher than that of group A, B and D (P < 0.01, P < 0.05, P < 0.05, respectively) (Figure 7j).

## Discussion

There was no significant correlation between data from HAA ELISA and IgA deposition area (Figure 3e). pIgA1 trap also revealed that there was no linear correlation between an aberrancy of *O*-glycan in serum pIgA1 and Area-IgA (Figure 3f). We hypothesized that IgAN patients have diversified profiles of *O*-glycan of serum IgA1 at the time of renal biopsy and that the patients with similar *O*-glycan profiles of serum IgA1 showed similar intensities of glomerular IgA deposition.

We showed the relationships among the three parameters including Area-IgA, data from HAA ELISA, and the pIgA1 trap in two dimensions by contour plots. This graphic representation suggested that patients with specific under-*O*-glycosylated profiles have similar clinical parameters including glomerular immunoglobulin and complement deposition levels and renal function. Area-IgA and serum IgA level were inversely distributed as well as Area-C3 and serum C3 level (Figure 4). It also seemed that patients with middle-level, HAA-ELISA titer values have the common characteristics of younger age, low-level IgA deposition, no IgG deposition, low C3 deposition, low urinary protein excretion, and good renal function. It seemed that these findings supported our hypothesis.

Decision tree analysis is commonly used for analysis of clinical data since it is flexible enough to express typical features of data such as nonlinearities and interactions [23]. The patients were roughly separated into 4 groups (A, B, C, D) with the HAA ELISA titer, and Group C was further divided into two groups (C1 and C2) with the pIgA1 trap value (Figure 5a, 5b). The pIgA1 trap value of groups A, B, and D had no significant differences (Figure 5c). In the overlaid border areas of the groups on the contour plot for Area-IgA, data from ELISA and the pIgA1 trap showed that patients with a higher level of Area-IgA were located in the area of Group B (Figure 5d) and overlaid borders on the other contour plots supported the reliability of the decision tree (data not shown).

The serum pIgA1 of IgAN patients is more elevated than in healthy controls [3] and peripheral lymphoid cells from IgAN patients synthesize significantly more pIgA1 than control subjects by pokeweed mitogen [24]. The glomerular IgA1 deposits were mainly polymeric [25,26]. Clearance kinetics and renal deposition analysis of soluble IgA immune complex revealed that the clearance of monomeric IgA immune complexes was more rapid than that of pIgA1 immune complexes and only pIgA1 immune complexes deposited in glomeruli [27]. The deglycosylated pIgA1 showed a significantly stronger binding capacity to human mesangial cells than native pIgA1, while deglycosylated mIgA did not bind to mesangial cells [28]. IgA1-IgG or IgA1-IgA immune complex formations in serum have critical roles in the glomerular immunoglobulin deposition of IgAN. This phenomenon was derived from over-production of aberrantly galactosylated IgA1 in the serum and the generation glycan-specific IgG and IgA autoantibodies [11,29-32].

The patients in groups A and B showed IgA1 and IgG co-deposition in glomeruli, suggesting IgA1-IgG immune complex formation in serum (Table 2). The complement system was activated since C3 deposition was evident while the serum C3 level was low [33,34]. Circulating IgA1-IgG immune complexes are associated with the activation of the complement system [35] and IgA1-glycan specific IgG immune complexes are elevated in IgAN patients, in addition to the levels urinary IgA1-glycan specific IgG immune complexes being correlated with proteinuria in IgAN [11]. Moreover, these immune complexes directly stimulate glomerular mesangial cells to produce C3 [36]. Thus, the patients in groups A and B have IgA1-IgG immune complex type IgAN with the activation of the complement system.

**Table 2:** Summary of decision tree analysis using HAA ELISA and pIgA1 trap

	**A**	**B**	**C1**	**C2**	**D**
HAA ELISA titer	+	++	+++	+++	++++
pIgA trap value	Middle	Middle	Middle	High	Middle
IgA deposition area	Middle	High	Low	Low	High
IgG deposition area	Middle	Middle	Low	Low	Low
C3 deposition area	Middle	High	Middle	Middle	Low
Age	Middle	Middle	High	Low	High
Urinary protein excretion	High	High	Low	Low	Middle
Hematuria	++++	++	+++	+	+++
Serum creatinine	High	High	Middle	Low	Middle
eGFR	Low	Low	Middle	High	Middle
Serum IgA	Low	Middle	Low	High	High
Serum C3	Low	Low	Low	Low	High
Histological grade	Middle	High	Middle	Low	High

Patients belonging to Group C (C1 and C2) were very heterogeneous in the deposition of IgA in glomeruli, as compared with other groups (Figure 6a). The characteristics of Group C were weak IgA deposition in glomeruli without IgG depositions although a middle-range HAA ELISA titer was observed (Figure 5b, 6a). The pIgA1 trap value was elevated in Group C2. Glomerular C3 deposition was strong and the serum C3 level was in the low range in Group C, which suggests an activated complement pathway and consumption. The pIgA1 trap value was elevated in Group C2 (Table 2). According to these findings, it was suggested that an immune complex consisting of under-*O*-glycosylated pIgA1 was dominant in sera in Group C2 patients. The age of the patients in Group C2 was younger than the other groups as well as having had a higher eGFR and mild histopathological finding in renal specimens (Figure 7a, d, and g).

Urinary protein excretion of group C2 became almost null three years after renal biopsy (Figure 7i) and eGFR three years after renal biopsy were higher than those at the time of renal biopsy (Figure 7j). These finding suggests that the disease activity of the patients in C2 group became calm but they were under hyperfiltration state reflecting the decrease of functional nephrons in the past. It was reported that serum IgA/C3 ratio was useful for predicting diagnosis in IgAN and positively correlates to severity of prognostic grading [33]. The mean IgA/C3 ratio of the patients in group C2 (3.72 ± 1.29) was highest of all the grpups (A: 2.84 ± 1.2, B: 3.36 ± 0.88, C1: 2.83 ± 1.18, D: 3.06 ± 1.60) but there were no significantly difference. While the mean IgA/C3 ratio of whole our patients was 3.13 ± 1.18, that of previous report whose samples were collected from 1980 to 1999 was 4.55 ± 1.21 [33]. Since our samples were collected in 2007 or 2008, the time of sample collection might induce decreasing IgA/C3 ratio. The decrease of IgA/C3 ratio was made by decrease of serum IgA. Mean serum IgA levels of our patients was much less than previous report (305.5 ± 127.1, 378.5 ± 106.4, respectively) [33]. Hygienic conditions or other unknown factors changed in these two or three decades might influence the IgA production in IgAN patients. Although serum IgA/C3 ratio is still useful, it is possibly limited to adopt IgA/C3 ratio for predicting the prognosis of recent IgAN patients.

Patents in Group D showed the highest HAA ELISA titer, a middle-range pIgA1 trap value, intense glomerular IgA deposition without IgG deposition, and severe renal histopathological findings. Being older in age, middle-ranged renal function and urinary protein excretion, and elevated serum IgA concentrations were also characteristic. The elevated C3 concentration in serum and weak C3 deposition in glomeruli were especially different from the other groups (Table 2). Since glomerular IgG deposition was not observed in this group, it was suggested that the immune complex consisted of under-*O*-glycosylated IgA1-glycan specific IgA immune complexes or self-aggregated under-*O*-glycosylated IgA1 [8]. Weak glomerular C3 deposition suggested that main component of this immune complex was monomeric IgA1 [37]. Although monomeric IgA or monomeric IgA immune complexes did not deposit into glomeruli, cross-linked monomeric IgA could deposit in glomeruli [27]. The induction of complement activation and glomerular deposition by administering cross-linked monomeric IgA was highly dependent on the nature of the antigen [38].

Table 3 shows a summary of the speculated immune complexes deposited in glomeruli and component consumption of the patients in each group.

**Table 3:** Speculated form of glomerular immune complex and complements consumption

**Group**	**A**	**B**	**C1**	**C2**	**D**
**Speculated Immune**	**IgA-IgG**	**IgA-IgG**	**Self-aggregated pIgA**	**Self-aggregated pIgA**	**Self-aggregated mIgA**
**complex**					**mIgA-mIgA**
Complements	↓	↓	↓	↓	→
Remarks				Early phase?	

## Conclusion

It is concluded that serum under-*O*-glycosylated IgA1 was not linearly correlated with glomerular IgA deposition in IgAN patients due to the heterogeneity in the composition of immune complexes of each patient.

## Abbreviations

IgAN: IgA nephropathy; ELISA: Enzyme-linked immunosorbent assay; HAA: *Helix aspersa*; pIgA1: Polymeric IgA1; mFcα/μR: Mouse Fcα/μ receptor; Area-IgA: Percentage area of IgA deposition in the whole glomeruli; GalNac: *N*-acetyl-*D*-galactosamine; poly-IgR: Polymeric immunoglobulin receptor; s-Cr: Serum creatinine; C3: Complement 3; HPF: High-power field; eGFR: Estimated glomerular filtration rate; BSA: Bovine serum albumin; PE: Phycoerythrin; MFI: Mean fluorescence intensity; WEKA: Waikato Environment for Knowledge Analysis.

## Competing interests

The authors declare that they have no competing interests.

## Authors’ contributions

KS and YS designed study, carried out HAA ELISA and pIgA1 trap, analyzed the data, and drafted the manuscript. YS helped in collecting samples. HY and HS were trained in HAA ELISA and helped performing ELISA. YS was a leader of this study group and gave suggestions for the study design. SH also gave suggestions and assisted in data analysis and interpretation. SH, KS and AS kindly presented mouse and human Fcα/μR transfectants and wonderful suggestions. YT organized this study. All authors have read and approved the final manuscript.

## Pre-publication history

The pre-publication history for this paper can be accessed here:

http://www.biomedcentral.com/1471-2369/15/89/prepub
